# Supplementary material for: Modanovo: A Unified Model for Post-translational Modification-Aware De Novo Sequencing Using Experimental Spectra From In Vivo and Synthetic Peptides
Source: Mol Cell Proteomics. 2025 Dec 24;25(2):101501. doi: 10.1016/j.mcpro.2025.101501 (PMC12860953; doi:10.1016/j.mcpro.2025.101501)
Supplement: Supplementary Data 1 [file mmc1.pdf]

Supplementary Information

Modanovo: A Unified Model for Post-Translational Modification-Aware de Novo Sequencing Using Experimental Spectra from In Vivo and Synthetic Peptides

Daniela Klaproth-Andrade[1], Yanik Bruns[1], Wassim Gabriel[2], Christian Nix[1], Valter Bergant[3,4], Andreas Pichlmair[3,5], Mathias Wilhelm[2,6,*], Julien Gagneur[1,6,7,8*]

[1] TUM School of Computation, Information and Technology, Technical University of Munich, Garching, Germany

[2] TUM School of Life Sciences, Technical University of Munich, Munich, Germany

[3] Institute of Virology, Technical University of Munich, School of Medicine, Munich, Germany

[4] National Institute of Chemistry, Ljubljana, Slovenia

[5] German Centre for Infection Research (DZIF), Partner site Munich, Munich, Germany

[6] Munich Data Science Institute (MDSI), Technical University of Munich, Garching, Germany

[7] Institute of Human Genetics, School of Medicine, Technical University of Munich, Munich, Germany

[8] Computational Health Center, Helmholtz Center Munich, Neuherberg, Germany

* To whom correspondence should be addressed.

Table 1: Post-translational modification (PTM)–amino acid combinations included in the development dataset. For each PTM, the amino acid that can contain this PTM, corresponding mass shifts (delta masses), and Unimod identifiers are provided, along with the source dataset(s) from which the modified peptides were obtained.

Modification	Residue(s)	Delta mass	Unimod Identifier	Source dataset(s)
N-term acetylation	-	+42.011 Da	1	MULTI-PTM, MassIVE-KB (v1)
Acetylation	Lysine (K)	+42.011 Da	1	MULTI-PTM
Phosphorylation	Serine (S),<br>Threonine (T),<br>Tyrosine (Y),	+79.966 Da	21	MULTI-PTM
Monomethylation	Arginine (R),<br>Lysine (K)	+14.016 Da	34	MULTI-PTM
Citrullination	Arginine (R)	+0.984 Da	7	MULTI-PTM
Ubiquitylation	Lysine (K)	+114.043 Da	121	MULTI-PTM
Oxidation	Methionine	+15.995 Da	35	MULTI-PTM, MassIVE-KB (v1)
Pyroglutamate formation	Glutamine (Q),<br>Glutamic acid (E)	-17.027 Da<br>-18.011 Da	28<br>27	MULTI-PTM
OGalNAc/OGlcNAc	Serine (S),<br>Threonine (T)	+203.079 Da	43	MULTI-PTM
N-term Carbamylation	-	+43.006 Da	5	MassIVE-KB (v1)
N-term ammonia loss	-	-17.027 Da	385	MassIVE-KB (v1)
Deamidation	Aspartic Acid (D),<br>Asparagine (Q)	+0.984 Da	7	MassIVE-KB (v1)

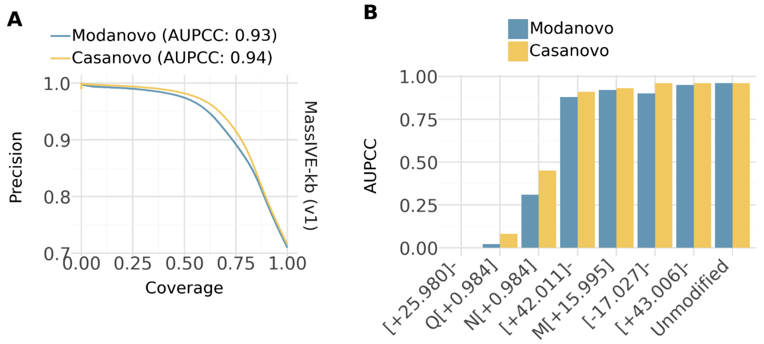

Supplementary Figure S1: Performance on the MassIVE-KB (v1) dataset compared to Casanovo. A, Precision-coverage curves at the peptide level comparing Modanovo (blue) to Casanovo (v4, yellow) on the test set of the MassIVE-KB (v1) dataset, originally used for training Casanovo, consisting mostly of unmodified peptide sequences. **B**, Area under the precision-coverage curve (AUPCC) obtained using Modanovo (blue) and Casanovo (v4, yellow) for the different PTM-residue combinations contained in the test set of MassIVE-KB (v1).

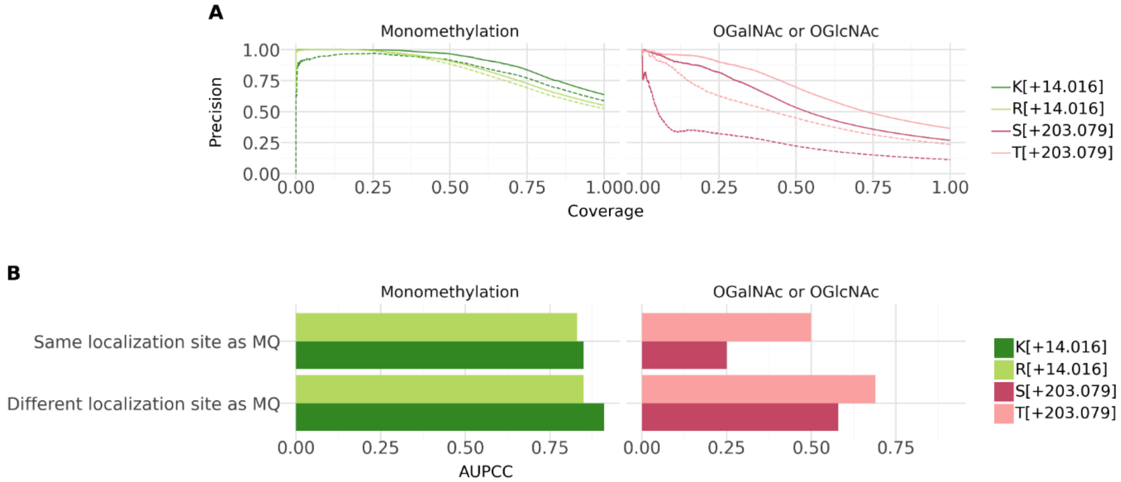

Supplementary Figure S2: Performance on monomethylation, O-GalNAc, and O-GlcNAc modifications allowing for different modification sites. A, Precision-coverage curves at the peptide level for the PTM types monomethylation and OGalNAc/OGlcNAc in the MULTI-PTM dataset. Curves are shown separately for lysine (K[+14.016]), arginine (R[+14.016]), serine (S[+203.079]), and threonine (T[+203.079]). Solid lines indicate cases where the predicted sequences are considered correct, even in cases where the predicted PTM localization site differs from the site reported by MaxQuant (MQ), while dashed lines indicate peptide correctness only when the same localization sites as MaxQuant were predicted. **B,** Area under the precision-coverage curve (AUPCC) for lysine (K[+14.016]), arginine (R[+14.016]), serine (S[+203.079]), and threonine (T[+203.079]) for cases where the predicted sequences are considered as correct, even in cases where the predicted PTM localization site differs from the site reported by MaxQuant (MQ), and for peptide correctness only when the same localization sites as MaxQuant were predicted.

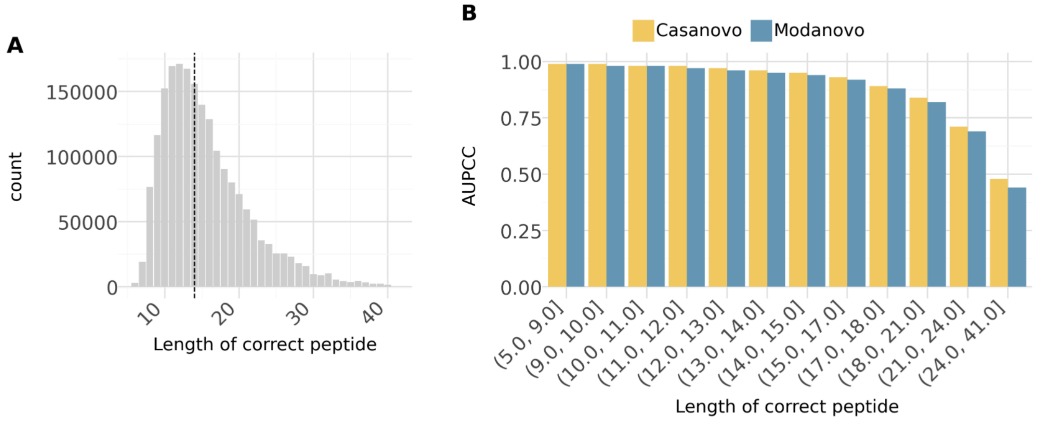

Supplementary Figure S3: Performance by peptide length on the MassIVE-KB (v1) dataset. A, Distribution of peptide lengths in the MassIVE-KB (v1) dataset. Dashed vertical lines indicate median peptide length. **B,** Area under the precision-coverage curve (AUPCC) for different peptide length categories obtained with Casanovo (yellow) and Modanovo (blue).

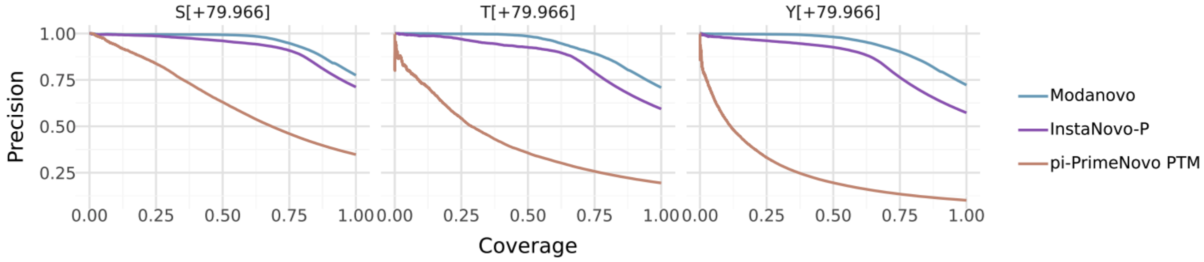

Supplementary Figure S4: Performance on phosphorylated peptides in comparison to π-PrimeNovo-PTM and InstaNovo-P. Precision-coverage curves at the peptide level for phosphorylation in the MULTI-PTM dataset. Curves are shown separately for serine (S[+79.966]), threonine (T[+79.966]), and tyrosine (Y[+79.966]) for Modanovo (blue) and the π-PrimeNovo-PTM model (brown) and InstaNovo-P (violet), which were fine-tuned to allow the prediction of phosphorylated residues.

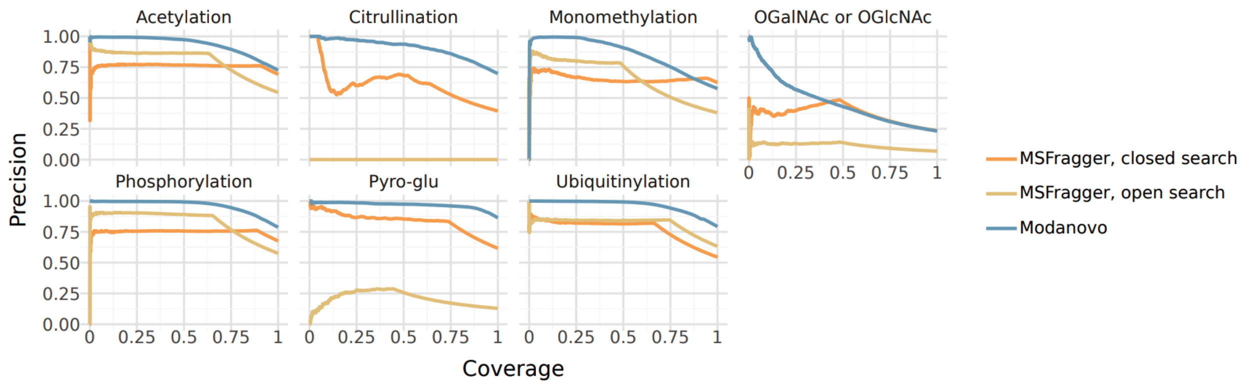

Supplementary Figure S5: Performance on the MULTI-PTM dataset compared to different MSFragger settings. Precision-coverage curves at the peptide level comparing Modanovo (blue) to MSFragger (claimed 1% FDR) in closed search mode (orange) and in open search mode (mustard) on the test set of the MULTI-PTM dataset, faceted by the different PTM types. MSFragger often does not propose a peptide for a given spectrum. These are ranked last and cause the hyperbole sections on the higher coverage range.

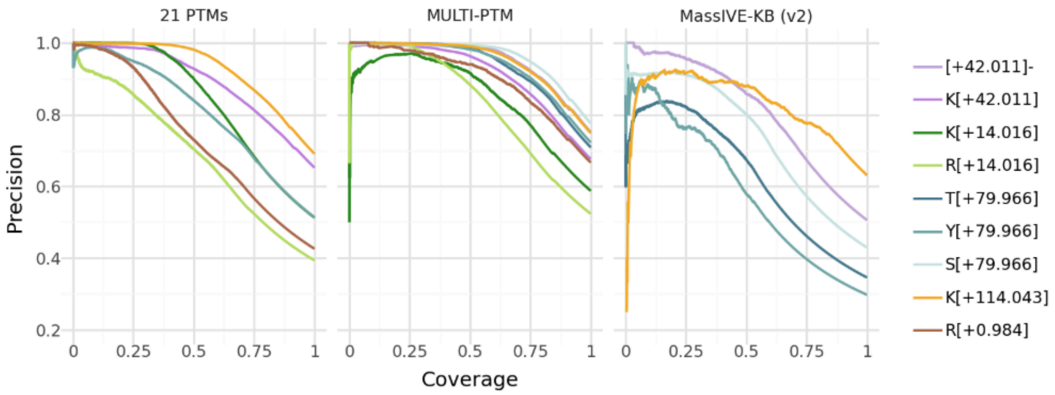

Supplementary Figure S6: Precision-coverage curves for Modanovo's predictions at the peptide level for the different PTM-residue combinations in the three different datasets, MassIVE-KB (v2), MULTI-PTM and 21 PTMs from ProteomeTools. PTM-residue combinations are restricted to those seen during model training and overlapping with those in MassIVE-KB (v2) and the 21-PTM dataset.

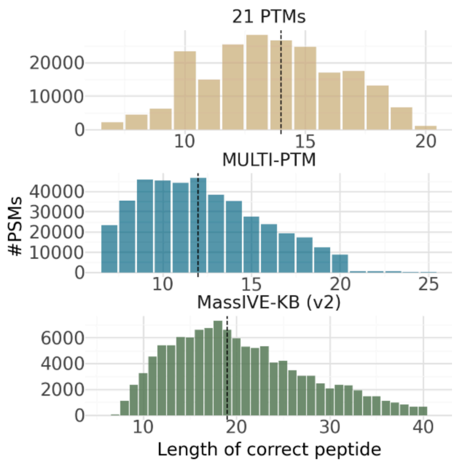

Supplementary Figure S7: Peptide length distribution in the datasets MassIVE-KB (v2) and 21 PTMs. The top panel shows the number of peptide-spectrum matches (PSMs) as a function of peptide length for the 21-PTM dataset (bottom panel), the MULTI-PTM dataset (middle panel), and the MassIVE-KB (v2) dataset (bottom panel). Dashed vertical lines indicate the median peptide length in each dataset. PSMs restricted to those carrying modifications seen during model training and overlapping with those in MassIVE-KB (v2) and the 21-PTM dataset.

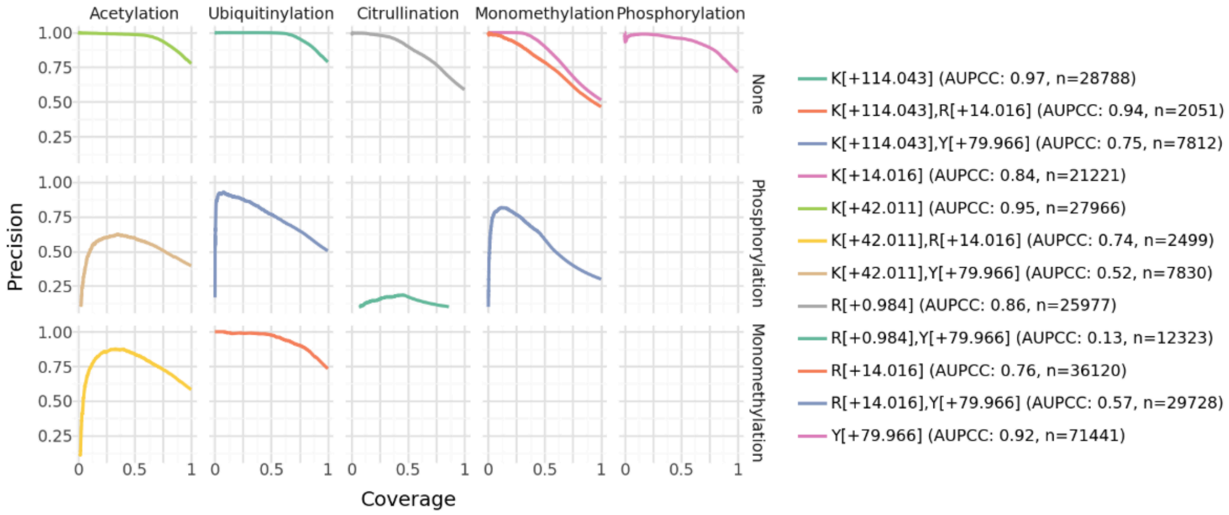

Supplementary Figure S8: Performance on the 21-PTM dataset, including peptides with multiple PTM types. Precision-coverage curves are shown for peptide sequences containing either a single PTM type (first row) or combinations of two PTM types (second and third rows; e.g., acetylation and phosphorylation in the second row, first column). Colored lines indicate specific amino acid-PTM combinations, with the corresponding area under the precision-coverage curve (AUPCC) reported in the legend.

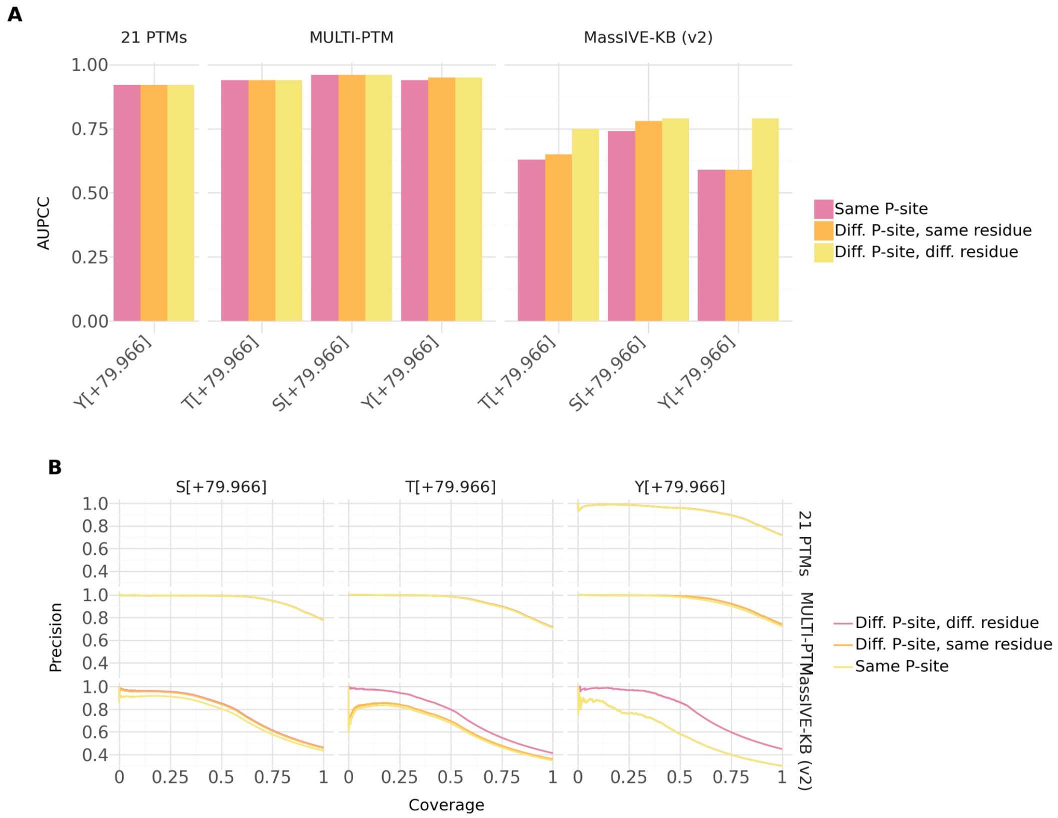

Supplementary Figure S9: A, Area under the precision-coverage curve (AUPCC) for the different phosphorylated residues on the MassIVE-KB (v2), MULTI-PTM and 21-PTM datasets, comparing against the same P-site as the ground truth peptide (rosa), allowing for a different P-site between Modanovo's predictions and the ground truth peptide on the same residue (orange) and allowing for a different P-site on a different residue (yellow). **B**, Same as A, but displaying the precision-coverage curves for the different residues and P-site considerations.

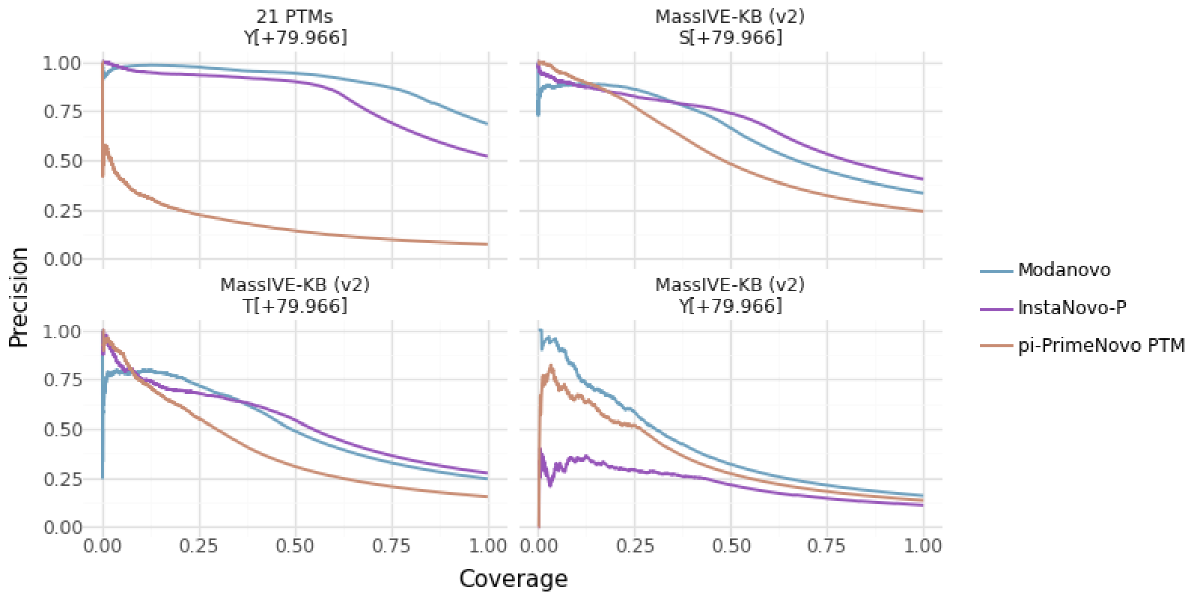

Supplementary Figure S10: Phosphorylation performance on the MassIVE-KB (v2) and 21-PTM datasets compared to π-PrimeNovo-PTM. Precision-coverage curves for phosphorylation on serine (S), threonine (T), and tyrosine (Y) residues (+79.966 Da) for Modanovo (blue) compared to π-PrimeNovo-PTM (brown) and InstaNovo-P (violet) fine-tuned to predict phosphorylated residues on the 21-PTMs and MassIVE-KB (v2) datasets (facets).

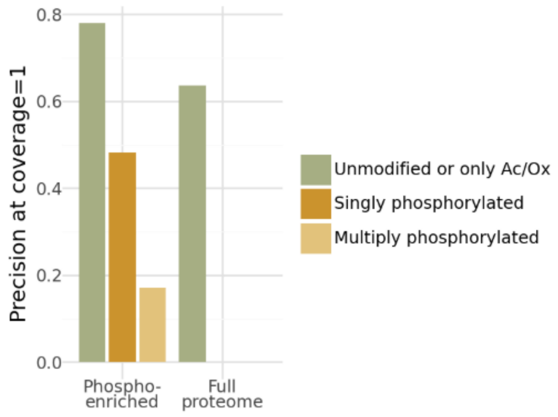

Supplementary Figure S11: Performance on the MPXV dataset compared to MaxQuant identifications. Final precision (at coverage=1) obtained by Modanovo with MaxQuant peptides as ground truth sequences for comparison for the full proteome samples and phosphorylation-enriched samples, with the MaxQuant sequence having multiple phosphorylated residues (light mustard), one phosphorylation residue (mustard), and no phosphorylation residues (green).

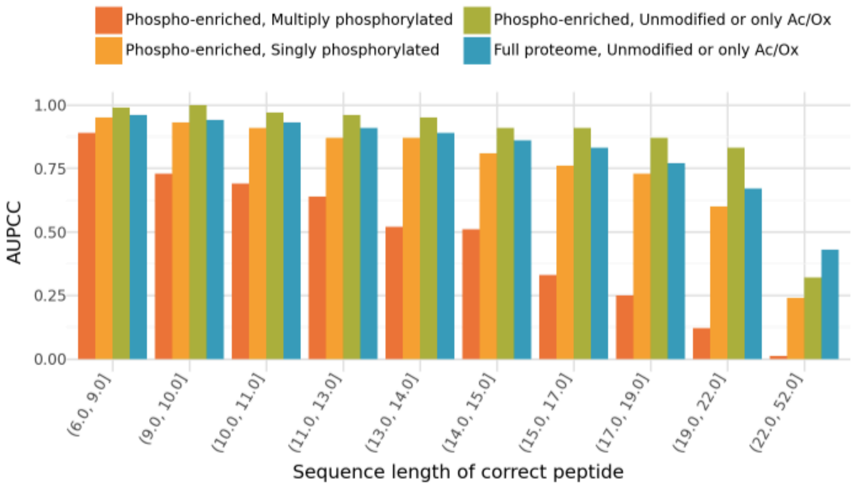

Supplementary Figure S12: Modanovo's performance on the MPXV dataset stratified by different peptide length categories. Area under the precision-coverage curve (AUPCC) by Modanovo on the MPXV dataset, stratified by peptide length category and modification status: multiply phosphorylated peptides (orange), singly phosphorylated peptides (yellow), unmodified peptides or those containing only N-terminal acetylation/oxidation in the phospho-enriched dataset (green), and unmodified peptides or those containing only N-terminal acetylation/oxidation in the full proteome dataset (blue).

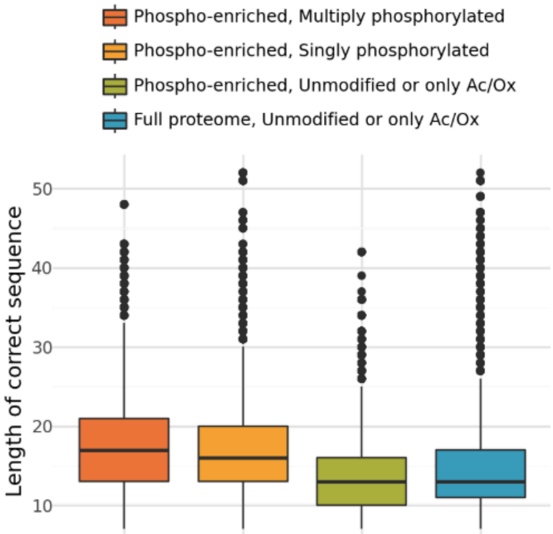

Supplementary Figure S13: Peptide length distributions of MaxQuant peptides on the MPXV dataset. Distribution of peptide lengths of the MaxQuant peptides in the MPXV dataset for multiply phosphorylated peptides (orange), singly phosphorylated peptides (yellow), unmodified peptides or those containing only N-terminal acetylation/oxidation in the phospho-enriched dataset (green), and unmodified peptides or those containing only N-terminal acetylation/oxidation in the full proteome dataset (blue).

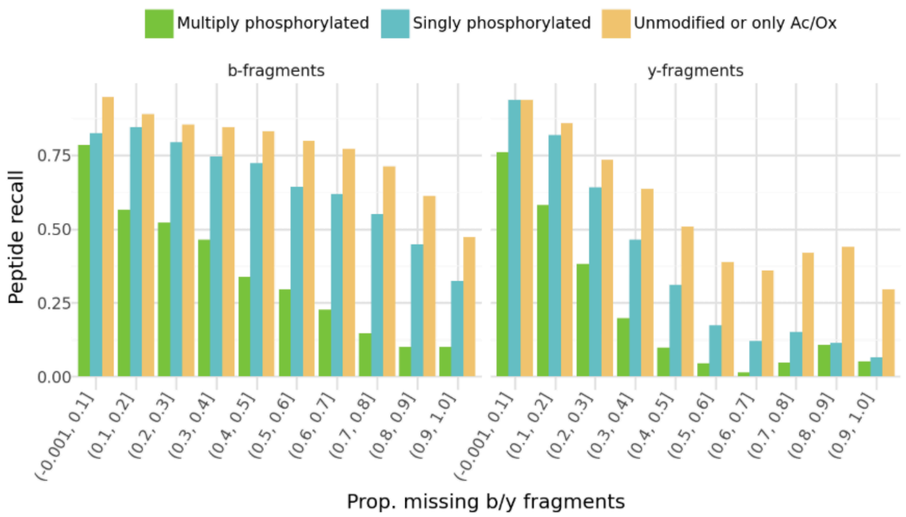

Supplementary Figure S14: Impact of fragment ion coverage on model performance in the MPXV dataset. Final precision (at coverage=1, i.e., peptide recall) as a function of the proportion of missing b-ions (left) and y-ions (right) in the spectra, stratified by peptide type: multiply phosphorylated (green), singly phosphorylated (blue), and unmodified or containing only N-terminal acetylation/oxidation (yellow) in the phospho-enriched samples of the MPXV dataset.

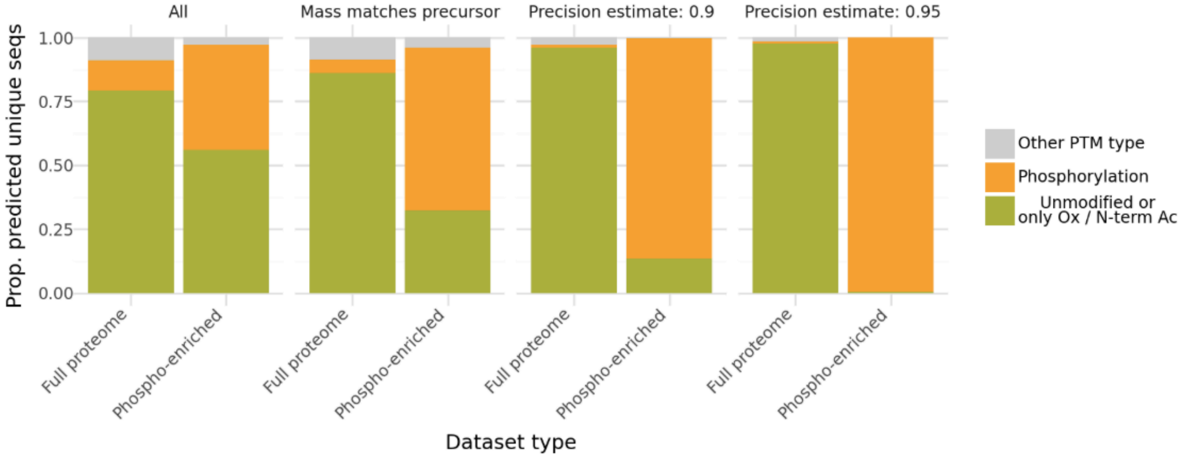

Supplementary Figure S15: Modanovo's predictions on the MPXV dataset. Proportion of predicted unique peptide sequences in the MPXV dataset by dataset type (full proteome vs. phospho-enriched) and different filtering criteria: all predictions, mass-matched to precursor, and subsets with estimated precision ≥0.9 and ≥0.95. Bars show the relative contribution of unmodified peptides or those with only oxidation/N-terminal acetylation (green), phosphorylated peptides (orange), and peptides with other PTM types (gray).

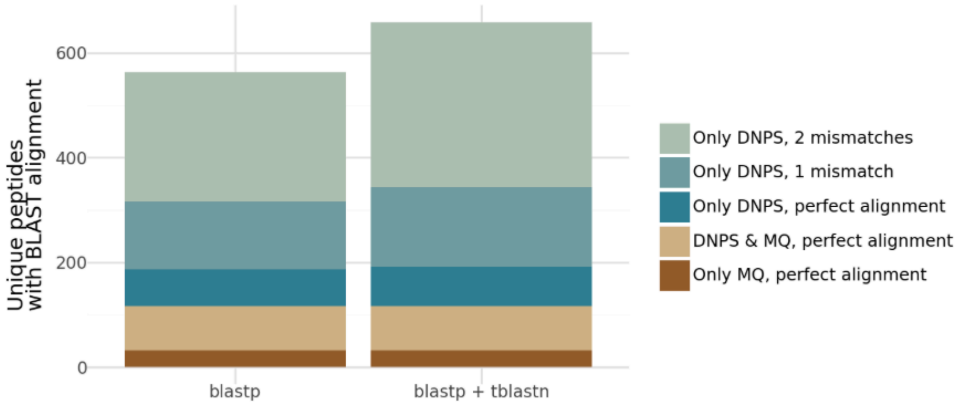

Supplementary Figure S16: Comparison of unique peptide identifications in the MPXV dataset based on BLAST alignment. Number of unique peptides recovered using blastp alone (left) or blastp combined with tblastn (right), stratified by source of identification: only DNPS with two mismatches (light green), only DNPS with one mismatch (teal-green), only DNPS with perfect alignment (blue), peptides identified by both DNPS and MaxQuant (MQ) with perfect alignment (beige), and only MQ with perfect alignment (brown).

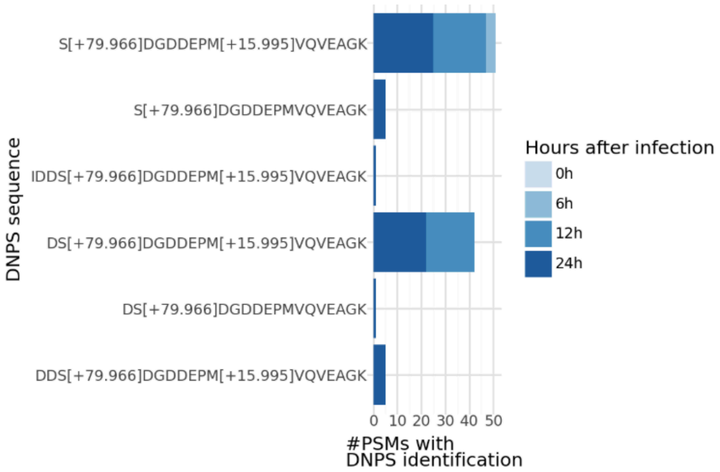

Supplementary Figure S17: Temporal detection of DNPS-identified peptides carrying the H5 phosphosite S116. Total number of PSMs identified for each peptide sequence containing the S116 phosphorylation site in the viral protein H5, separated by infection time point (0, 6, 12, and 24h).

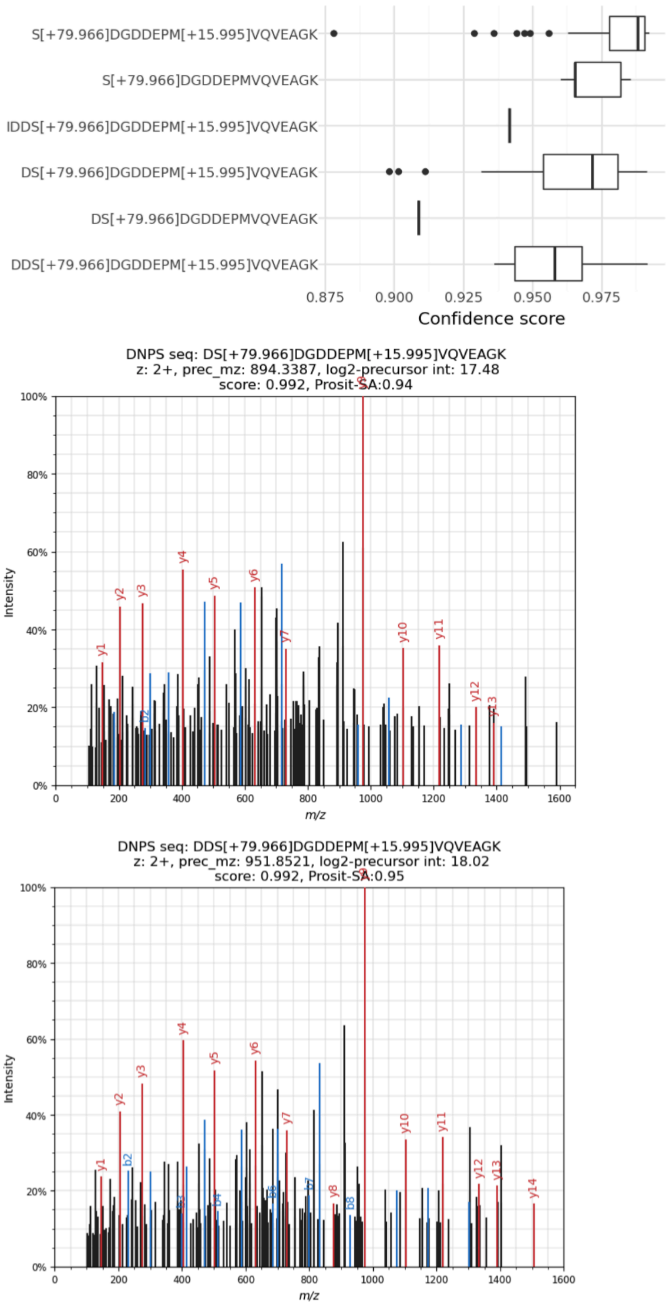

Supplementary Figure S18: MS2 spectral evidence for the Modanovo-identified phosphosite S116 in H5. A, Modanovo's confidence scores for the six distinct peptide sequences carrying the S116 phosphorylation site in the viral protein H5, each supported by multiple PSMs. **B,** Example annotated spectrum for the peptide DS[+79.966]DGDDEPM[+15.995]VQVEAGK, illustrating fragment ion coverage around the phosphorylated serine. **C,** Same as B, but for the peptide sequence DDS[+79.966]DGDDEPM[+15.995]VQVEAGK.
